# The adaptive role of the knee joint in maintaining the orbital stability during slope walking

**DOI:** 10.1098/rsos.252138

**Published:** 2026-09-09

**Authors:** Jungho Lee, Chae Lynne Kim, Ilseung Park, Jeongin Moon, Jooeun Ahn

**Affiliations:** ^1^ Department of Physical Education, Seoul National University, Seoul, Republic of Korea; ^2^ Department of Mechanical Engineering, Carnegie Mellon University, Pittsburgh, PA, USA; ^3^ Department of Mechanical and Industrial Engineering, University of Massachusetts Lowell, Lowell, MA, USA; ^4^ Institute of Sport Science, Seoul National University, Seoul, Republic of Korea

**Keywords:** gait stability, nonlinear dynamics, slope walking, orbital gait stability

## Abstract

Maintaining gait stability on sloped surfaces is a biomechanically demanding task. Previous studies revealed the effect of slopes on overall gait stability, but the joint-level mechanisms underlying the stability still remain unclear. Given that most active exoskeletons for gait assistance focus on providing additional torque to specific joints without considering the contribution of each joint to the stability, understanding the role of each joint in stabilization can enable the design of a safer intervention. In this study, we aimed to identify joint-specific contributions to gait stability by analysing maximum Floquet multipliers (max FMs) and their corresponding eigenvector across multiple gait phases and slope conditions. Results were obtained from the data of 13 participants walking on a treadmill at five slope gradients (−12°, −6°, 0°, 6° and 12°). The max FMs remained mostly invariant across slopes and phases, whereas the eigenvector components of the bilateral knees showed significant phase- and slope-dependent changes. These variations exhibited alternating patterns between limbs and were moderately correlated with joint angle variability, highlighting the knee's adaptive role in maintaining stability. Our findings provide new insights into joint-level stabilization strategies, which could potentially serve as valuable biomechanical insight for future exoskeleton design. Moreover, our findings suggest that interference at the knee during slope walking could largely affect gait orbital stability relative to other lower limb joints.

## 1. Introduction

Humans maintain robust stability when walking in diverse environments, such as various inclines and declines [1]. Walking on sloped surfaces is a daily activity that significantly increases demand on the locomotor system and raises the risk of falls [2,3], so maintaining a stable gait on slopes is a common challenge [4,5]. Walking on slopes alters gait biomechanics compared with level walking, changing posture [6,7], muscle activity [8,9], kinematic patterns [10–12] and joint kinetics [4,5,13], all of which influence gait stability [1,14–16].

Previous studies have investigated the effects of sloped environments on stability [1,14–16]. Studies revealed that whole-body angular momentum in the sagittal plane is tightly regulated during downhill walking to maintain balance and prevent excessive forward momentum, and this regulation becomes increasingly difficult at steeper slopes [1,17]. Similarly, gait variability and the maximal Lyapunov exponent increased as slopes became steeper [14]. These results indicate that steeper slopes lead to reduced overall gait stability and increase difficulty in maintaining balance. Thus, the slope gradient may change how individual joints contribute to overall gait stability. However, such joint-level mechanisms behind gait stability in sloped walking remain largely unknown.

Floquet multipliers (FMs) have been used to quantify gait orbital stability [18–21]. FM quantifies the rate of a system's convergence to a periodic limit cycle after a perturbation [18]. Previous studies have observed how people manage to maintain an orbitally stable gait despite the destabilizing characteristics, and how humans respond differently to perturbations given at different gait phases [22–24]. Although these studies revealed that humans are able to maintain orbital stability throughout the gait phases under perturbation, they did not reveal how such stability could be managed.

One of the noteworthy features of FM is that it is an eigenvalue of a matrix that describes the error dynamics; FM always accompanies the corresponding eigenvector. In particular, the eigenvector corresponding to the FM with the largest magnitude (max FM) has the information of the mode of the most destabilizing perturbation. A previous study found that a three-dimensional passive dynamic walking model exhibited an unstable mode in the roll motion using FMs and the corresponding eigenvectors [25]; the unstable motion was primarily dominated by the roll angle and velocity, which were identified as the eigenvector components with the largest magnitudes. This modelling study further revealed that the control of lateral foot placement could enable the stabilization of the roll motion. Motivated by this insight from simple walking models, multiple studies have investigated human walking and suggested the important role of the active control of medial-lateral foot placement in stabilizing level walking [26,27]. Such active control is also crucially required during sloped walking, in which joint angle variability is adjusted according to slope gradient to maintain overall stability [10]. Therefore, identifying the eigenvector corresponding to the max FM during slope walking can contribute to understanding the stabilizing mechanism or the mode of the most destabilizing perturbations on slopes.

One of the applications that urgently needs such understanding of the stabilizing mechanism in various walking environments is the gait-assistive exoskeleton. Partly owing to the global ageing of the population, interest in gait-assistive exoskeletons is increasing rapidly [28,29]. However, most active exoskeletons have focused only on adding power to specific joints uniformly [30–35], whether the user walks on level, declined or inclined surfaces [13]. Blind use of such exoskeletons without understanding the effect of slopes on the role of each joint in stabilizing the gait might jeopardize gait stability, which should take on a higher priority than efficiency in power generation obtained by the assistance [36–38]. Identifying joint-level contributions to gait stability can lead to specifically targeted interventions for gait stabilization and safer design and application of assistive exoskeletons.

However, to the best of our knowledge, no study has examined the eigenvector components corresponding to the max FM in human gait to investigate how each joint individually contributes to gait stability in various environments. Therefore, we aim to scrutinize joint-level stabilizing mechanisms in human walking under various conditions. Specifically, we quantify whether and how the max FM and its corresponding eigenvector vary across multiple gait phases and slopes. We further investigate whether the eigenvector components and the variability of joint kinematics are correlated under each gait condition.

## 2. Methods

### 2.1. Participants

Based on a previous study [39], we set the effect size as 0.5, with a statistical power and significance level of 0.95 and 0.05, respectively. Given the effect size, power and significance, G*Power software (Heinrich Heine University, Düsseldorf, Germany) computed the required sample size as 12. Accordingly, we recruited 13 healthy young adult males (age: 21.4 ± 2.1 years, height: 1.77 ± 0.05 m, mass: 74 ± 5.6 kg) in the study. The participants underwent a preliminary screening and had no known history of cardiovascular, muscular, orthopaedic or neurological diseases. The study received approval from the Institutional Review Board of Seoul National University on 26 November 2024 (IRB number 2410/001-012). All participants were thoroughly informed about the study and its procedures, and written consent was obtained prior to the start of the experiment.

### 2.2. Experimental protocol and set-up

Prior to the main experiment, the preferred walking speed (PWS) of each participant was calculated using the up-down method following previous research [40]. Participants walked on a treadmill and were instructed to identify a speed that most accurately approximated their habitual walking pace. Starting at 2.5 km h^−1^, the treadmill speed was promptly increased by 0.1 km h^−1^ at 10-s intervals until the participant reported a speed that felt closest to their natural walking speed. Then, the treadmill speed was increased by 1.0 km h^−1^ and subsequently reduced by 0.1 km h^−1^ at 10-s intervals until the participant once again confirmed a speed that felt closest to their natural walking speed. Given that variations in PWS could influence the experimental outcomes, the measurement process was repeated three times, from which the average speed was calculated as the participant's PWS [40].

In the main experiment, each participant walked on a split-belt instrumented treadmill under five different slope conditions (−12°, −6°, 0°, 6° and 12°) at a fixed speed set to 80% of their determined PWS (0.91 ± 0.08 m s^−1^). Slope conditions were provided in randomized order, while three repeated trials under the same slope condition were conducted consecutively with a 3-minute rest period between trials. Motion and force data were collected for 5 minutes during each of the 15 trials.

### 2.3. Measurements

Fifty-four reflective markers were placed across the participants’ head, torso, arms, pelvis, lower limbs and feet, following the Rajagopal marker set [41]. Kinematic data were captured using a nine-camera, marker-based motion capture system (Qualisys, Sweden; 100 Hz), and kinetic data were recorded using a split-belt instrumented treadmill (Bertec Corp, USA, 1000 Hz). Prior to each experimental session, the motion capture system was calibrated to achieve a tracking residual of less than 0.5 mm.

### 2.4. Data analysis

A zero-lag low-pass Butterworth filter with a cut-off frequency of 10 Hz was applied to filter the raw coordinates of the reflective markers. We followed the coordinate system recommended by the International Society of Biomechanics, where the *X*, *Y* and *Z* axes correspond to the medial–lateral, anterior–posterior and vertical directions of the retro-reflective markers, respectively. A static trial was conducted prior to the walking trials, where participants were instructed to stand in the standard anatomical position for 5 s. Using this static trial data, a seven-segment lower limb model consisting of the pelvis, thighs, shanks and feet was created on biomechanical modelling software (Visual3D v6^TM^, C-Motion Inc., Maryland, USA), later used to calculate the kinematics of the lower limb joints.

#### 2.4.1. Gait event detection

**Figure 1.**
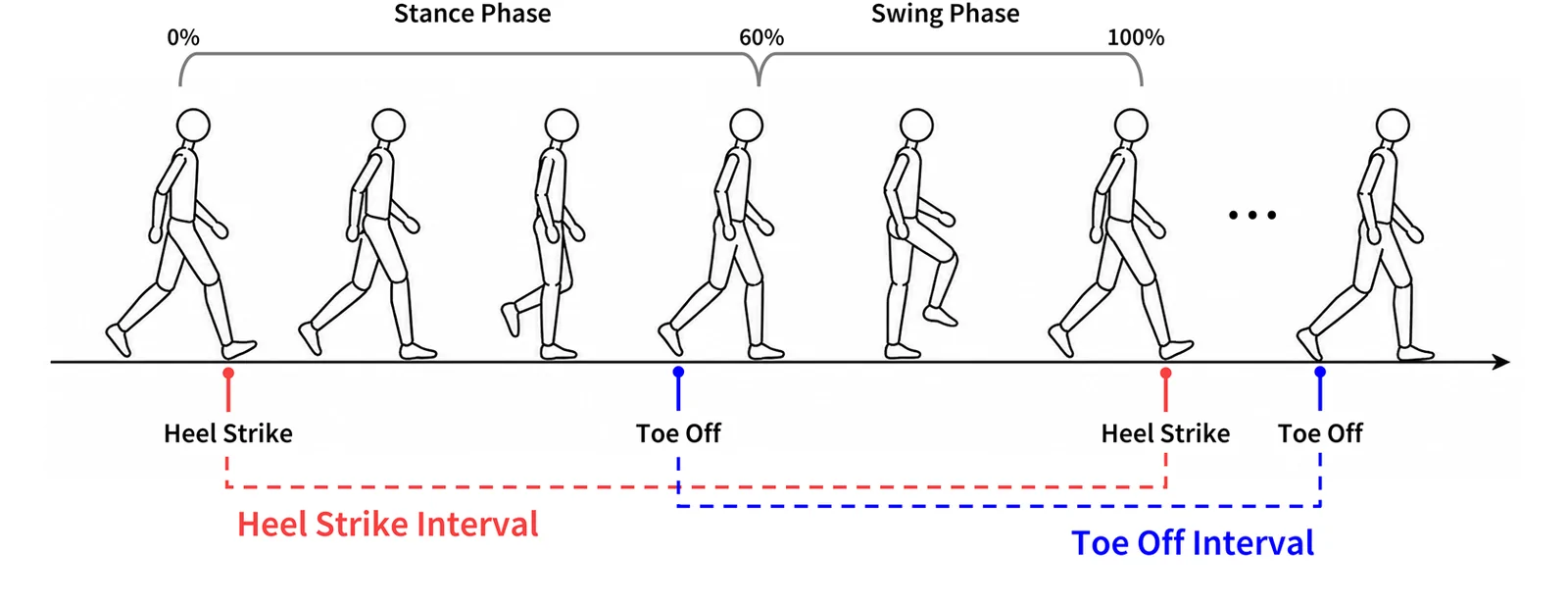
Illustration of gait cycles defined by gait events. Gait cycles were defined as intervals between the HS of one foot and the next HS of the same foot, or as intervals between the TO of one foot and the next TO of the same foot. Depending on each subject's leg dominance, gait cycles were defined as intervals between subsequent dominant/non-dominant leg HSs, and subsequent dominant/non-dominant leg TOs.

As illustrated in figure 1, we defined one gait cycle as the interval from the heel strike (HS) of one foot to the subsequent HS of the same foot and from the toe off (TO) of one foot to the subsequent TO of the same foot. The HS and TO events were identified using the distance between the anterior–posterior position of the pelvis centre of mass and the heel marker. The HS was identified as the time point corresponding to a local maximum of this distance, whereas the TO was identified as the time point corresponding to a local minimum [42].

#### 2.4.2. Orbital stability

To assess orbital stability, we computed FM using a time series analysis method, well established in previous studies [18,43]. We first constructed a time series of 14-dimensional state vectors, which included the three degrees of freedom (DOF) angles of the hip, the flexion/extension of the knee, and the three DOF angles of the ankle for both limbs. As the sample size of 200 strides is considered the minimum length of time series required for a reliable calculation of FM [44], we constructed state vectors from 200 strides. We anchored the Poincaré sections at HSs and TOs of both limbs—dominant leg HS (DHS), non-dominant leg HS (NDHS), dominant leg TO (DTO) and non-dominant leg TO (NDTO). This allowed us to construct a Poincaré map where the inputs and outputs are 14-dimensional vectors originating from each gait event of the preceding stride and its following stride, respectively. Expressed in equation (2.1), *F* represents the Poincaré map, and *S_k_* refers to the state vector at the *k*th cycle.

(2.1) $$S_{k+1}\;=\;F\left(S_{k}\right).$$

In theory, during periodic motion, the input or output of the Poincaré map, denoted as S*, becomes a fixed point of the map and consistently satisfies the condition (equation (2.2)).

(2.2) $$S^{*}\;=\;F\left(S^{*}\right).$$

In human gait data, where various sources of noise and perturbation exist, the exact S^*^ is unknown. Therefore, the average of the state vector is typically used as an approximation of S^*^ [39]. Abiding by this conventional process, we selected S^*^ as the average joint state vector at the gait event of all extracted strides for each Poincaré section. After determining *S*^∗^, we constructed a time series of error vectors by subtracting S^*^ from each *S_k_*. Then we established a linear map that closely approximated the relationship between errors across successive cycles (equation (2.3)):

(2.3) $$\;\left[S_{k+1\;}-S^{*}\right]=\;J\left[S_{k}-S^{*}\right],$$

where *J* represents the Jacobian matrix, estimated using the least squares error method. We then calculated the largest eigenvalue of the obtained Jacobian matrix, the max FM, at four Poincaré sections (DHS, NDHS, DTO and NDTO) in five slope conditions (−12°, −6°, 0°, 6° and 12°). The max FM serves as an indicator of orbital stability of the defined system, with values ranging from 0 to 1. The max FM values closer to 0 indicate rapid convergence to the limit cycle and thus stronger orbital stability, whereas values closer to 1 indicate slow convergence and therefore weaker orbital stability.

#### 2.4.3. Eigenvector corresponding to the max FM

We analysed the 14-dimensional eigenvectors corresponding to the max FM at each of the four Poincaré sections in five slope conditions. Since components of eigenvectors are complex numbers in general, we analysed their magnitudes to quantify the relative contribution of each joint DOF to the most unstable mode [25]. For the eigenvector corresponding to the max FM, the components with larger magnitudes represent the variable whose error is more likely to jeopardize the stability. In essence, each eigenvector component represents the contribution of its corresponding joint's DOF to the direction of the most destabilizing perturbation.

#### 2.4.4. Joint angle variability

We analysed the variability of joint angles in the hip, knee and ankle, each corresponding to components consisting of the state vectors constructed for the calculation of FM. We calculated the average standard deviation (mean s.d.) of each joint angle across 200 strides at each Poincaré section [10].

### 2.5. Statistics

The mean and standard error of the max FM were computed for each trial. A two-way repeated measures analysis of variance (ANOVA) was used to compare the max FM with five slope conditions and four Poincaré sections. The mean and standard error of eigenvector component magnitudes corresponding to the max FM were computed for each trial. A two-way repeated measures ANOVA was conducted to compare the eigenvector components corresponding to the max FM with five slope conditions and four Poincaré sections. Mauchly's test of sphericity was performed to evaluate the sphericity assumption for each variable. When the assumption was violated, the Greenhouse–Geisser correction was applied to adjust the degrees of freedom. Post hoc pairwise comparisons were conducted using the Bonferroni test. Statistical significance was set at *p* < 0.05. Pearson's correlation (*r*) was used to examine relationships between eigenvector component magnitudes and joint angle variability as well as between contralateral eigenvector components. All statistical tests were conducted with jamovi (The jamovi project, 2025).

## 3. Results

### 3.1. Maximum Floquet multipliers

**Figure 2.**
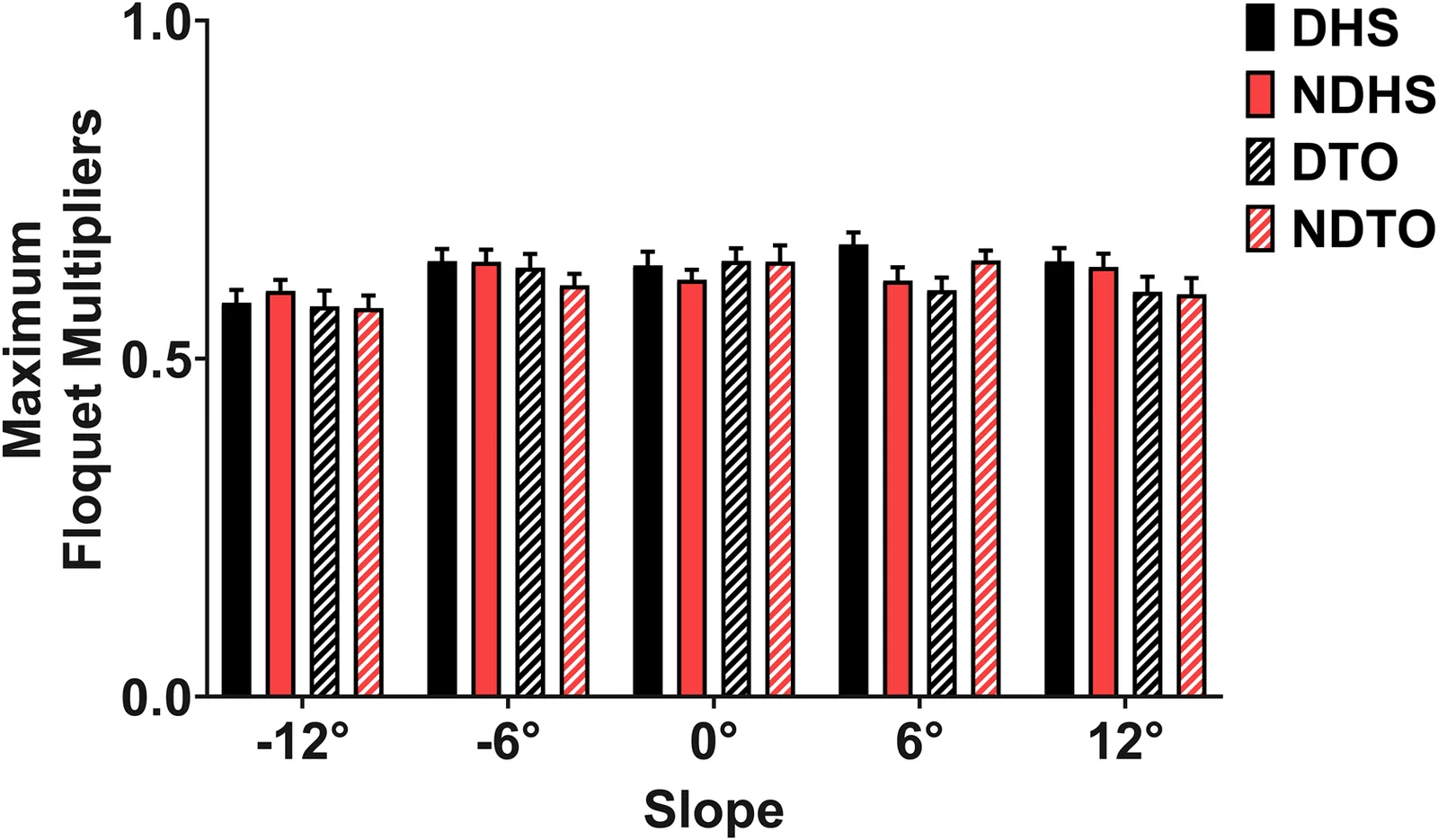
Mean and standard error of max FM at Poincaré sections across slope conditions. The bars represent the mean values of max FM, and the error bars represent the standard error. DHS, NDHS, DTO and NDTO, respectively, represent dominant leg heel strike, non-dominant leg heel strike, dominant leg toe off and non-dominant leg toe off.

The mean and standard error of the max FM across all slope conditions at each Poincaré section are illustrated in figure 2. A two-way repeated measures ANOVA with Greenhouse–Geisser correction revealed a significant main effect of Poincaré section (*F*_(2.62, 31.42)_ = 4.66, *p* = 0.011, η^2^_p_ = 0.280) and a significant interaction effect between Poincaré section and slope (*F*_(5.85, 70.25)_ = 3.47, *p* = 0.005, η^2^_p_ = 0.224). However, slope conditions did not show a significant main effect on max FM (*F*_(2.80, 33.64)_ = 2.77, *p* = 0.06, η^2^_p_ = 0.188). According to Bonferroni post hoc pairwise comparisons, neither the main effect of slope nor the interaction between Poincaré section and slopes was significant, whereas DHS and DTO Poincaré sections showed significant differences (*t*_(12)_ = 4.59, *p* = 0.004). The mean and standard deviation of max FM at each Poincaré section across slopes are summarized in table 1.

**Table 1.** Mean ± s.d. of max FM at Poincaré sections across slope conditions. DHS, NDHS, DTO and NDTO, respectively, represent dominant leg heel strike, non-dominant leg heel strike, dominant leg toe off and non-dominant leg toe off.

		**slope**				
		−12°	−6°	0°	6°	12°
**Poincaré section**	**DHS**	0.582 ± 0.069	0.644 ± 0.066	0.638 ± 0.074	0.669 ± 0.064	0.644 ± 0.071
	**NDHS**	0.600 ± 0.060	0.643 ± 0.066	0.616 ± 0.053	0.615 ± 0.072	0.635 ± 0.072
	**DTO**	0.577 ± 0.084	0.634 ± 0.075	0.644 ± 0.068	0.601 ± 0.068	0.599 ± 0.081
	**NDTO**	0.574 ± 0.069	0.608 ± 0.061	0.643 ± 0.086	0.645 ± 0.054	0.595 ± 0.087

### 3.2. Eigenvector component magnitudes

**Figure 3.**
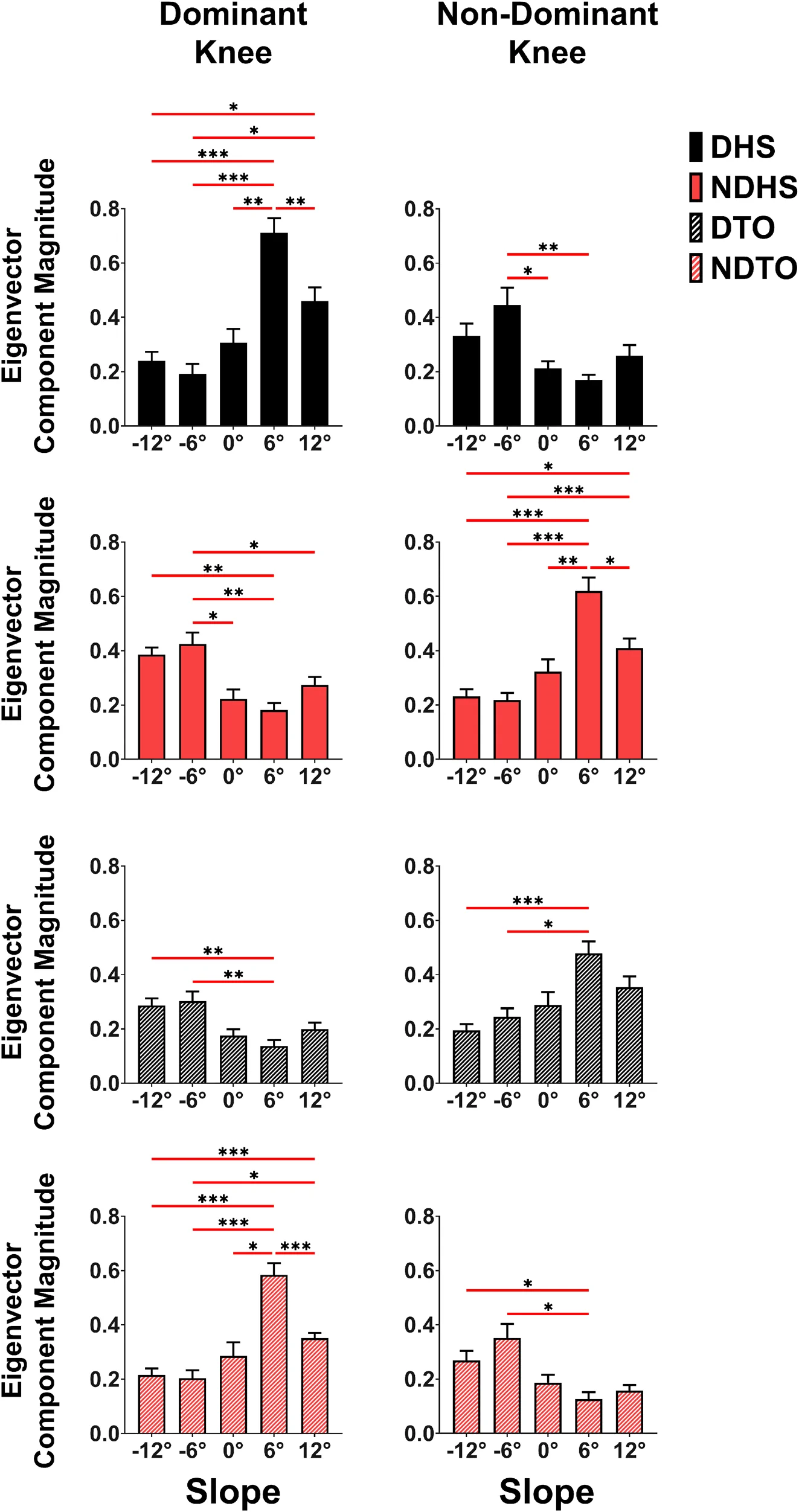
Mean and standard error of the magnitudes of the eigenvector components corresponding to the dominant leg and non-dominant leg knee's sagittal DOF. Each bar represents the mean value of the magnitude of the sagittal knee motion component of the eigenvector corresponding to the max FM at each Poincaré section across slope conditions, and the error bars represent the standard error. DHS, NDHS, DTO and NDTO, respectively, represent dominant leg heel strike, non-dominant leg heel strike, dominant leg toe off and non-dominant leg toe off. Asterisks denote statistically significant differences between slope conditions from Bonferroni post hoc pairwise comparisons (* *p* < 0.05, ** *p* < 0.01, *** *p* < 0.001).

The magnitudes of each component of the eigenvector corresponding to the max FM at each Poincaré section and slope condition were compared using two-way repeated measures ANOVA with the Greenhouse–Geisser correction. Eigenvector component magnitudes of all 14 DOFs across the lower limb joints were examined as part of our analysis, among which the knee showed particularly noteworthy findings. Out of the 14 eigenvector components corresponding to the max FM at each Poincaré section (supplementary material, table S2), significance in all effects, that is, main effect of slope, main effect of Poincaré section and interaction effect, was revealed for the components of the sagittal knee angle of both legs. Moreover, the eigenvector component of the sagittal knee angle showed the largest change in magnitude both across slope conditions and across Poincaré sections. Figure 3 shows the magnitudes of the dominant leg and non-dominant leg knee's sagittal components across slope conditions at each Poincaré section. A two-way repeated measures ANOVA concluded a significant main effect of slope on the dominant leg knee's sagittal component (*F*_(2.39, 28.70)_ = 9.52, *p* < 0.001, η^2^_p_ = 0.442) and non-dominant leg knee's sagittal component (*F*_(2.90, 34.84)_ = 4.69, *p* = 0.008, η^2^_p_ = 0.281). The main effect of Poincaré section was also concluded as significant on the dominant leg (*F*_(2.76, 33.12)_ = 17.40, *p* < 0.001, η^2^_p_ = 0.592) and the non-dominant leg knee's sagittal component (*F*_(2.80, 33.62)_ = 12.27, *p* < 0.001, η^2^_p_ = 0.506). Furthermore, the interaction effect between slope and Poincaré section showed significance in the dominant leg knee (*F*_(4.30, 51.54)_ = 19.15, *p* < 0.001, η^2^_p_ = 0.615) and the non-dominant leg knee's sagittal component (*F*_(5.29, 63.49)_ = 12.30, *p* < 0.001, η^2^_p_ = 0.506), respectively. Bonferroni post hoc pairwise comparisons revealed significant differences between −12° and 6° slopes for the magnitudes of the eigenvector component of both the dominant leg knee (*t*_(12)_ = −5.174, *p* = 0.002) and the non-dominant leg knee (*t*_(12)_ = −4.728, *p* = 0.005). As for comparisons between Poincaré sections, the dominant leg knee's sagittal components were shown to be significantly different between the DHS-NDHS (*t*_(12)_ = 3.44, *p* = 0.03), DHS-DTO (*t*_(12)_ = 7.15, *p* < 0.001), NDHS-DTO (*t*_(12)_ = 4.30, *p* = 0.006) and NDTO-DTO (*t*_(12)_ = 4.61, *p* = 0.004) phases and the non-dominant leg knee's sagittal component magnitudes were significantly different between DHS-NDHS (*t*_(12)_ = −3.23, *p* = 0.043), NDHS-NDTO (*t*_(12)_ = 5.34, *p* = 0.001) and NDTO-DTO (*t*_(12)_ = −3.81, *p* = 0.015) phases.

**Figure 4.**
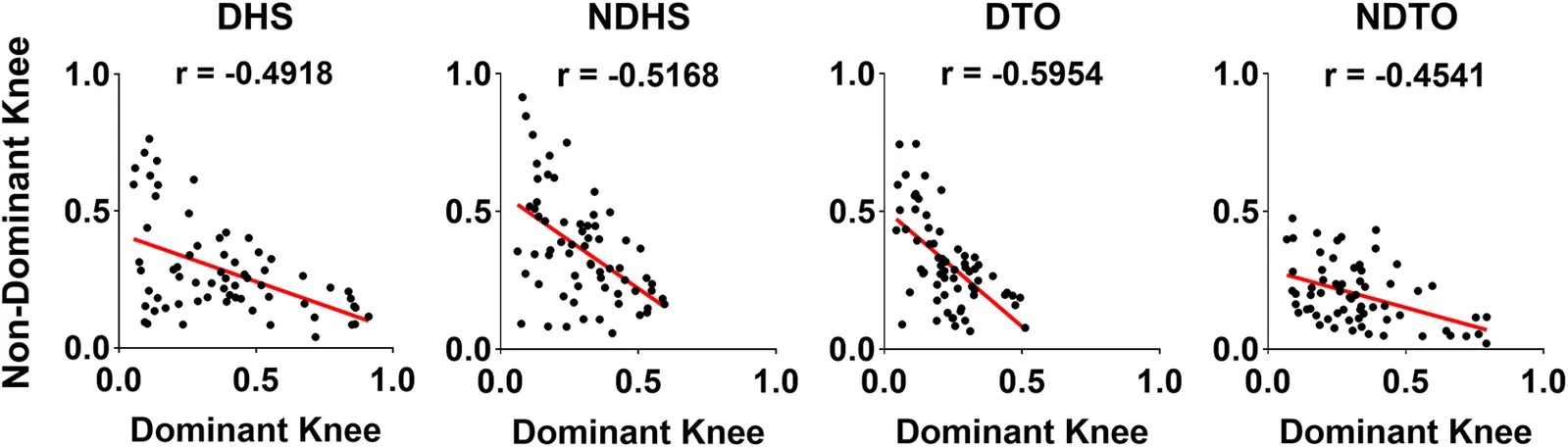
Correlation between the magnitudes of the sagittal components of the dominant leg knee (*x*-axis) and the non-dominant leg knee (*y*-axis) in the eigenvector corresponding to the max FM. DHS, NDHS, DTO and NDTO, respectively, represent dominant leg heel strike, non-dominant leg heel strike, dominant leg toe off and non-dominant leg toe off.

To further investigate inter-limb relationships for stabilization, we conducted a Pearson correlation analysis between the magnitudes of the sagittal components of the dominant leg and non-dominant leg knees in the eigenvector corresponding to the max FM. As illustrated in figure 4, the magnitudes of the sagittal components of the dominant leg and non-dominant leg knees showed a negative correlation with moderate to strong strength (DHS: *r* = −0.49, *p* < 0.001 / NDHS: *r* = −0.52, *p* < 0.001 / DTO: *r* = −0.60, *p* < 0.001 / NDTO: *r* = −0.45, *p* < 0.001) in all Poincaré sections.

### 3.3. Joint angle variability

**Figure 5.**
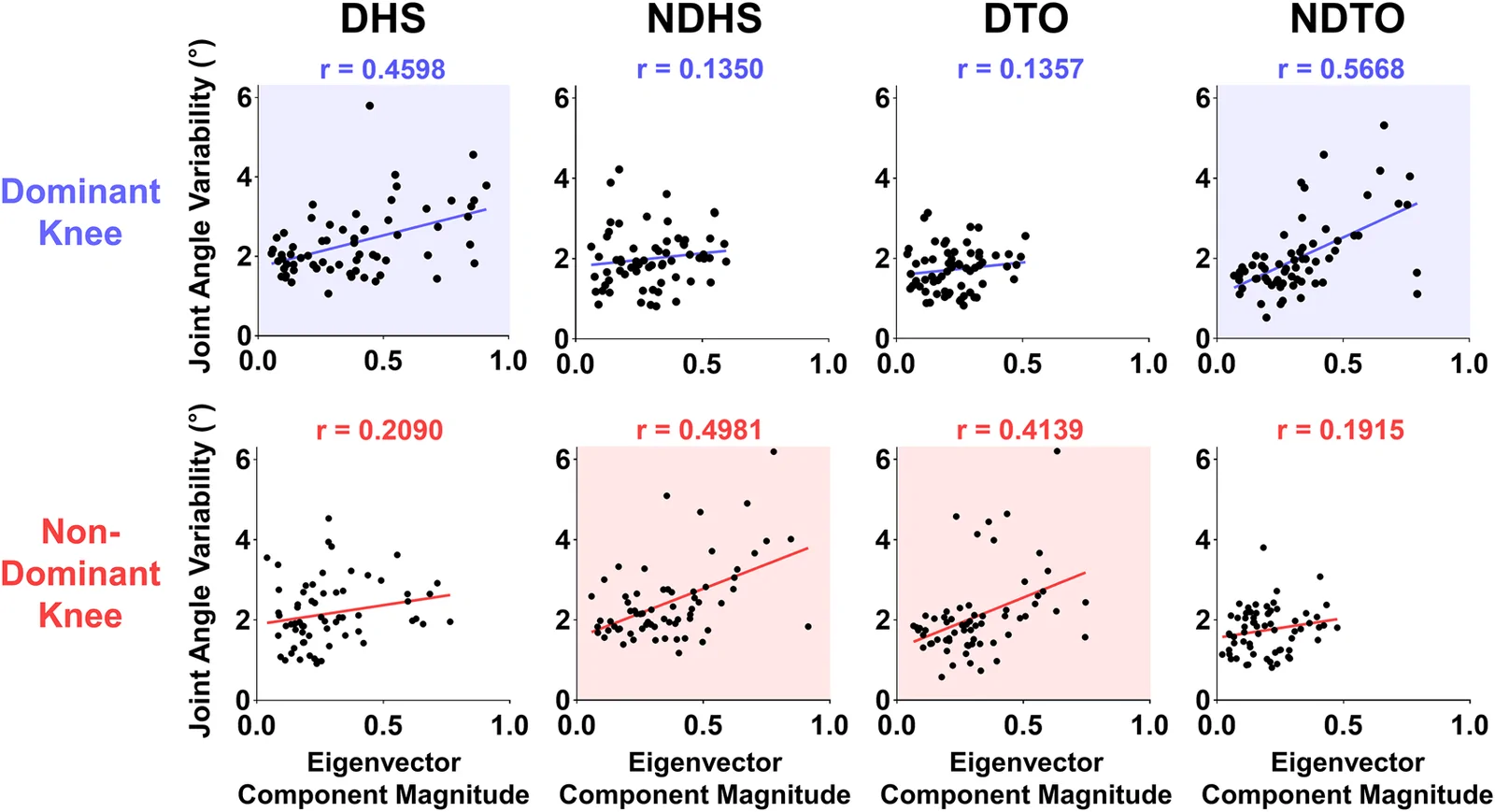
Correlation between eigenvector component magnitudes and joint angle variability at each Poincaré section for the sagittal dominant and non-dominant knee, respectively. Each dot represents the mean value of a subject at a given gait phase and slope condition. Shaded subplots indicate statistical significance in correlation.

We conducted a Pearson correlation analysis between the magnitude of eigenvector component and joint angle variability as shown in figure 5. The dominant leg knee's sagittal eigenvector component magnitude and its sagittal joint angle variability had positive correlations with moderate strength at the DHS (*r* = 0.46, *p* < 0.001) and NDTO (*r* = 0.57, *p* < 0.001) phases, respectively. Conversely, the non-dominant leg knee's sagittal eigenvector component magnitude and its sagittal joint angle variability had positive correlations with moderate strength at the NDHS (*r* = 0.50, *p* < 0.001) and DTO (*r* = 0.41, *p* < 0.001) phases, respectively. Correlation at the NDHS (*r* = 0.14, *p* = 0.284) and DTO (*r* = 0.14, *p* = 0.281) phases was revealed as not significant for the dominant leg knee, and correlation at the DHS (*r* = 0.21, *p* = 0.095) and NDTO (*r* = 0.19, *p* = 0.126) was revealed as not significant for the non-dominant leg knee.

## 4. Discussion

Maintaining stable gait in sloped environments is a challenging yet prevalent task in daily life. Numerous studies have put forth the biomechanical changes owing to slopes and have evaluated their effects on local and orbital stability [1–18]. However, no study has revealed the joint-level vulnerability and contribution in relation to orbital stability. Moreover, a study investigating the alterations in joint-level vulnerability across gait phases in sloped conditions is unprecedented. In this study, we analysed the components of eigenvectors corresponding to the max FM at multiple gait phases and evaluated the correlation between an individual joint's contribution to stability and its angle variability. Although lower limb kinematics across slope conditions (supplementary material, table S1) were consistent with previous studies [12,45], the max FM showed a statistical difference only between the dominant leg HSs and dominant leg TOs in the post hoc test, with a minimal mean difference of 0.0222 (supplementary material, table S4). The reported mean difference of 0.0222 can be interpreted as minimal considering the entire range (i.e. between 0 and 1) of possible max FM values. Moreover, it is a notably small value when compared with the mean differences revealed as statistically significant between population groups in a previous study [46]. These results, however, do not agree with the previous study suggesting that the orbital stability of human walking is phase‑dependent [18]. Such a discrepancy could be attributed to the difference in walking environment, consequently yielding a different level of perturbation. Previous studies were limited to experimentally quantifying the locomotor system's response to the small perturbations that occur naturally during level walking. By contrast, our results were obtained in a slope environment, which makes locomotion relatively challenging compared with level walking. Our results suggest that sloped conditions may enforce a behaviour of strictly maintaining stability, causing the orbital stability of walking to be phase‑independent. A previous study demonstrated that the regulation of whole-body angular momentum becomes more difficult at high inclines and declines [17], and it is suggested that adopting a more stable locomotion strategy is preferred when the locomotor system is unable to exploit the beneficial effects of inertia [39]. The slope walking strategies observed in our study represent a case where regulating the posture during locomotion and taking advantage of the beneficial effects of inertia is challenging. Comprehensively, our findings suggest that healthy humans largely maintain a consistent level of orbital stability regardless of gait phase or gradient when walking on slopes between −12° and 12° at the speed comfortable for inclined walking.

As opposed to the sustaining of orbital stability, the eigenvector components of the bilateral knee joints in the sagittal plane exhibited an interchanging pattern across the gait phases depending on the slope environment. In uphill conditions, the magnitude of the dominant leg knee's sagittal eigenvector component increased in the dominant leg HSs and non-dominant leg TOs, whereas the magnitude of the corresponding component of the non-dominant leg knee increased in the non-dominant leg HSs and dominant leg TOs. By contrast, the magnitude of the dominant leg knee's sagittal eigenvector component increased in the non-dominant leg HSs and dominant leg TOs, whereas the magnitude of the corresponding component of the non-dominant leg knee increased in the dominant leg HSs and non-dominant leg TOs. These results suggest that when walking uphill, a perturbation applied on the knee timed at the HSs of the ipsilateral leg and TOs of the contralateral leg could crucially destabilize the gait. Conversely, when walking downhill, a perturbation applied at the HSs of the contralateral leg and TOs of the ipsilateral leg could jeopardize gait stability. Therefore, it is plausible that applying adequate assistance on the knee's sagittal motion at the specified gait phase and slopes could effectively lessen one's burden in maintaining gait stability. Our results also highlight the potential danger that applying assistance to the locomotor system, without considering its influence on stability, could act as a double-edged sword. Based on our findings, when developing gait-assistive exoskeletons, taking into account the alterations in joint-specific vulnerability along with the initial biomechanical advantages that the gait-assistive exoskeleton sought to achieve would better ensure locomotor stability.

Furthermore, a negative correlation between the eigenvector component magnitudes of bilateral knees in the sagittal DOF was observed (figure 3), suggesting that the dominant leg and non-dominant leg knee's respective contribution to stability has an inverse relationship throughout the gait cycle in various slope severities. The observed negative correlation suggests an inter-limb coordination strategy in maintaining stability; when one knee is more dominant in contributing to stability, the other knee contributes less. These findings are consistent with the results of previous studies revealing that the trailing leg performed considerable positive work during uphill walking, while the leading leg exhibited significant negative work during downhill walking [47]. Absorption of energy or negative work is essential for stability, and walking on slopes induces a shift in the roles of power generation and absorption among the bilateral lower limbs, allowing adaptation to sloped environments [47]. Likewise, the negative correlation between the magnitudes of eigenvector components of bilateral knees suggests a shift in the joint that dominantly contributes to orbital stability.

By assessing the correlation between the magnitude of eigenvector component and joint angle variability at different gait phases, we were able to quantify at which gait phases a joint's angle variability was related to the joint's contribution to stability. We observed that the knee joint, in particular, showed a positive correlation between the magnitude of eigenvector component and joint angle variability at the ipsilateral HSs and contralateral TOs. This may indicate that the knee serves a dynamically adaptive role at the specified gait events, possibly modulating the joint's contribution to gait stability during the transition of gait phases. Our findings align with previous studies demonstrating that individuals with episodic knee instability exhibit increased knee joint motion variability compared with their control counterparts, and that increased sagittal-plane knee motion variability is associated with a higher risk of falls in patients with knee osteoarthritis [48,49]. Such evidence underscores the importance of knee joint dynamics in maintaining balance and stability during complex locomotor tasks.

Our results extend understanding of gait stability on inclined and declined surfaces by exploiting the multi-dimensional information obtained from the eigenvector corresponding to max FM. The obtained results can provide a basis for identifying critical phases and joints associated with an increased risk of falls during slope walking. Our findings provide foundational biomechanical insight that may potentially extend to inform gait-assistive exoskeleton design. To ensure that gait-assistive exoskeletons provide safe interventions, it is essential not only to apply external torques [30–35] but also to consider orbital stability and the phase- and joint-specific contributions to the stability across various slope environments. Specifically, according to our findings, stabilizing the knee joints should be considered for walking on slopes.

This study has multiple limitations. First, we used a constant walking speed across all slope conditions. Since max FMs are influenced by walking speed [19,39,50], we provided a constant walking speed, aiming to examine only the effect of slopes on gait stability. Nevertheless, self-selected walking speeds may differ between uphill, downhill and level walking conditions [51]. Thus, self-selected walking speeds on slopes may induce different stabilization mechanisms. Hence, our results may possibly include the artefact effects owing to the unnaturally set and constrained walking speed. Future study that further examines the effect of slope on the stabilization mechanisms with the PWS under each slope condition is encouraged. Second, our findings are specific to young and healthy male adults, and may not be fully generalizable to older adults, females, patients, populations with pathological gait conditions, or other populations at a greater risk of falls. Thus, future study is necessary to investigate the underlying stabilization mechanisms in more various groups, particularly including individuals at a high risk of falls. Third, as highlighted in previous literature [52], it remains challenging to determine the most appropriate set of biomechanical variables to construct the state space for quantifying the orbital stability of human gait. Therefore, consulting multiple previous studies [39,43,53–56], we chose to use the lower limb kinematics and adopted a method proven to reduce errors in estimating the max FM [43]. Nevertheless, it is noteworthy that the methodology of our study is neither unique nor an ideal method in measuring the orbital stability in slope walking. Lastly, we constructed only the knee flexion–extension DOF in the state vector. Therefore, frontal- and transverse-plane knee motions were not considered in the stability analysis. These knee motions may contribute more to knee instability during real-world falls than sagittal-plane motion. Thus, future study is needed to include frontal- and transverse-plane knee motions in the state vector.

## 5. Conclusion

We extend understanding of gait stability on inclined and declined surfaces by exploiting the multi-dimensional information obtained from the eigenvector corresponding to max FM. Further, our findings suggest that healthy humans maintain a consistent level of orbital stability regardless of gait phase or slope gradient but adopt phase- and slope-dependent strategies with interchanging joint contributions to stabilization to achieve the consistent level of orbital stability. The obtained results can provide a basis for identifying critical phases and joints associated with an increased risk of falls during slope walking, and thus improving rehabilitation strategies and gait-assistive exoskeletons.

## Supplementary Material



## Data Availability

All experimental data and codes and supplementary materials supporting the findings of this study are publicly available through a Dryad Digital Repository [57]. Supplementary material is available online [58].
