# Supplementary material for: The adaptive role of the knee joint in maintaining the orbital stability during slope walking
Source: R Soc Open Sci. 2026 Sep 9;13(9):rsos252138. doi: 10.1098/rsos.252138 (PMC13561039; doi:10.1098/rsos.252138)
Supplement: Supplementary file 1 [file rsos.252138.zip › rsos.252138/rsos252138_si_001.docx]

**Supplementary**

**Table S1. Mean and standard deviation of range of motion (ROM) and minimum to maximum angles in lower limb joints aggregated from strides defined by subsequent dominant leg heel strikes across slope conditions.**

***Mean±SD (Min – Max)**

|  | **Slope** | | | | |
| --- | --- | --- | --- | --- | --- |
| **Joint ROMs** | **-12°** | **-6°** | **0°** | **6°** | **12°** |
| **Non-Dominant Ankle Sagittal** | 33.08 ± 2.52 (-13.81 – 19.26) | 31.36 ± 4.89 (-12.61 – 18.75) | 31.58 ± 3.71 (-14.49 – 17.09) | 36.04 ± 4.41 (-15.45 – 20.58) | 39.32 ± 5.00 (-13.69 – 25.63) |
| **Non-Dominant Ankle Frontal** | 14.18 ± 2.70 (-12.51 – 1.66) | 13.94 ± 3.98 (-12.33 – 1.61) | 11.71 ± 3.24 (-11.25 – 0.47) | 9.19 ± 1.61 (-10.30 – -1.10) | 8.73 ± 2.17 (-10.99 – -2.26) |
| **Non-Dominant Ankle Transverse** | 6.95 ± 1.91 (-10.30 – -3.35) | 7.71 ± 2.36 (-10.34 – -2.62) | 6.71 ± 1.47 (-9.93 – -3.21) | 7.19 ± 1.82 (-8.89 – -1.70) | 7.68 ± 2.32 (-8.17 – -0.49) |
| **Non-Dominant Hip Sagittal** | 19.20 ± 2.75 (11.17 – 30.36) | 28.07 ± 4.02 (3.71 – 31.79) | 37.90 ± 3.13 (-1.88 – 36.02) | 48.22 ± 3.44 (-1.15 – 47.07) | 59.38 ± 5.41 (-0.02 – 59.36) |
| **Non-Dominant Hip Frontal** | 11.43 ± 2.74 (-4.37 – 7.07) | 12.48 ± 2.59 (-5.25 – 7.24) | 12.14 ± 2.42 (-4.70 – 7.44) | 11.58 ± 2.53 (-4.95 – 6.63) | 12.04 ± 3.21 (-5.25 – 6.79) |
| **Non-Dominant Hip Transverse** | 12.63 ± 2.76 (-2.65 – 9.99) | 12.50 ± 2.93 (-2.76 – 9.74) | 11.53 ± 2.06 (-1.55 – 9.98) | 10.64 ± 1.89 (-2.25 – 8.39) | 11.02 ± 1.98 (-3.21 – 7.82) |
| **Non-Dominant Knee Sagittal** | 73.35 ± 4.50 (-78.79 – -5.44) | 68.23 ± 5.42 (-71.85 – -3.63) | 65.31 ± 4.24 (-69.11 – -3.80) | 55.46 ± 4.28 (-66.29 – -10.83) | 56.59 ± 5.26 (-67.27 – -10.68) |
| **Dominant  Ankle Sagittal** | 32.43 ± 3.24 (-13.08 – 19.35) | 29.90 ± 3.80 (-11.53 – 18.37) | 31.71 ± 3.37 (-15.38 – 16.32) | 35.45 ± 3.56 (-15.48 – 19.97) | 39.16 ± 4.40 (-13.55 – 25.61) |
| **Dominant  Ankle Frontal** | 16.57 ± 3.53 (-1.29 – 15.28) | 15.94 ± 3.84 (-0.94 – 14.99) | 12.93 ± 3.56 (0.44 – 13.37) | 9.54 ± 1.39 (2.29 – 11.83) | 8.80 ± 1.26 (3.67 – 12.47) |
| **Dominant  Ankle Transverse** | 7.04 ± 1.83 (0.36 – 7.40) | 7.42 ± 1.53 (-0.03 – 7.39) | 6.77 ± 1.71 (-0.12 – 6.66) | 7.21 ± 1.92 (-1.68 – 5.53) | 8.09 ± 2.09 (-3.30 – 4.79) |
| **Dominant Hip Sagittal** | 19.08 ± 2.51 (10.34 – 29.42) | 28.28 ± 3.36 (2.55 – 30.83) | 37.38 ± 2.69 (-2.33 – 35.05) | 47.97 ± 2.96 (-1.57 – 46.40) | 59.10 ± 4.92 (0.04 – 59.14) |
| **Dominant Hip Frontal** | 11.52 ± 2.23 (-3.68 – 7.84) | 12.53 ± 2.67  (-4.54 – 7.99) | 12.77 ± 3.12 (-4.99 – 7.78) | 12.75 ± 3.01 (-4.55 – 8.21) | 13.48 ± 3.50 (-4.99 – 8.50) |
| **Dominant Hip Transverse** | 12.74 ± 3.10 (-8.76 – 3.98) | 12.74 ± 2.67 (-9.04 – 3.70) | 11.58 ± 2.30 (-8.88 – 2.70) | 10.90 ± 1.47 (-7.47 – 3.42) | 11.18 ± 1.55 (-7.01 – 4.17) |
| **Dominant Knee Sagittal** | 72.57 ± 4.60 (-77.13 – -4.56) | 68.02 ± 5.05 (-70.95 – -2.93) | 65.56 ± 4.64 (-68.55 – -2.99) | 55.27 ± 5.03 (-65.56 – -10.29) | 55.09 ± 4.97 (-66.46 – -11.38) |

**Table S2. Mean and standard deviation of 14-dimensional eigenvector component magnitudes corresponding to the maximum Floquet multiplier at each Poincaré section across slope conditions.**

|  |  | **Slope** | | | | |
| --- | --- | --- | --- | --- | --- | --- |
| **Eigenvector Component** | **Poincaré  Section** | **-12°** | **-6°** | **0°** | **6°** | **12°** |
| **Non-Dominant Ankle Sagittal** | **DHS** | 0.156 ± 0.061 | 0.235 ± 0.091 | 0.117 ± 0.054 | 0.130 ± 0.061 | 0.165 ± 0.125 |
|  | **NDHS** | 0.276 ± 0.106 | 0.258 ± 0.194 | 0.211 ± 0.112 | 0.169 ± 0.081 | 0.203 ± 0.092 |
|  | **DTO** | 0.370 ± 0.153 | 0.300 ± 0.152 | 0.194 ± 0.076 | 0.297 ± 0.104 | 0.260 ± 0.112 |
|  | **NDTO** | 0.272 ± 0.095 | 0.276 ± 0.122 | 0.250 ± 0.106 | 0.294 ± 0.117 | 0.351 ± 0.157 |
| **Non-Dominant Ankle Frontal** | **DHS** | 0.109 ± 0.039 | 0.122 ± 0.045 | 0.163 ± 0.090 | 0.075 ± 0.031 | 0.128 ± 0.059 |
|  | **NDHS** | 0.174 ± 0.060 | 0.187 ± 0.077 | 0.178 ± 0.117 | 0.098 ± 0.052 | 0.102 ± 0.045 |
|  | **DTO** | 0.189 ± 0.103 | 0.212 ± 0.110 | 0.193 ± 0.076 | 0.091 ± 0.053 | 0.084 ± 0.043 |
|  | **NDTO** | 0.147 ± 0.083 | 0.145 ± 0.054 | 0.178 ± 0.112 | 0.103 ± 0.053 | 0.131 ± 0.076 |
| **Non-Dominant Ankle Transverse** | **DHS** | 0.061 ± 0.032 | 0.062 ± 0.030 | 0.118 ± 0.052 | 0.048 ± 0.028 | 0.085 ± 0.043 |
|  | **NDHS** | 0.107 ± 0.049 | 0.144 ± 0.075 | 0.121 ± 0.072 | 0.057 ± 0.036 | 0.081 ± 0.046 |
|  | **DTO** | 0.104 ± 0.050 | 0.147 ± 0.087 | 0.140 ± 0.053 | 0.074 ± 0.042 | 0.083 ± 0.047 |
|  | **NDTO** | 0.083 ± 0.054 | 0.066 ± 0.034 | 0.104 ± 0.061 | 0.085 ± 0.041 | 0.118 ± 0.059 |
| **Non-Dominant  Hip Sagittal** | **DHS** | 0.291 ± 0.110 | 0.245 ± 0.121 | 0.240 ± 0.080 | 0.229 ± 0.166 | 0.337 ± 0.160 |
|  | **NDHS** | 0.259 ± 0.097 | 0.190 ± 0.095 | 0.267 ± 0.078 | 0.250 ± 0.112 | 0.242 ± 0.122 |
|  | **DTO** | 0.229 ± 0.097 | 0.224 ± 0.135 | 0.251 ± 0.108 | 0.287 ± 0.075 | 0.304 ± 0.123 |
|  | **NDTO** | 0.250 ± 0.125 | 0.252 ± 0.128 | 0.224 ± 0.070 | 0.235 ± 0.125 | 0.300 ± 0.164 |
| **Non-Dominant  Hip Frontal** | **DHS** | 0.154 ± 0.079 | 0.151 ± 0.075 | 0.192 ± 0.131 | 0.098 ± 0.059 | 0.142 ± 0.089 |
|  | **NDHS** | 0.154 ± 0.070 | 0.151 ± 0.088 | 0.125 ± 0.048 | 0.115 ± 0.071 | 0.166 ± 0.050 |
|  | **DTO** | 0.134 ± 0.076 | 0.113 ± 0.048 | 0.163 ± 0.079 | 0.126 ± 0.071 | 0.183 ± 0.058 |
|  | **NDTO** | 0.156 ± 0.080 | 0.170 ± 0.059 | 0.193 ± 0.095 | 0.120 ± 0.051 | 0.130 ± 0.067 |
| **Non-Dominant  Hip Transverse** | **DHS** | 0.241 ± 0.138 | 0.249 ± 0.110 | 0.273 ± 0.113 | 0.175 ± 0.070 | 0.224 ± 0.060 |
|  | **NDHS** | 0.290 ± 0.125 | 0.213 ± 0.106 | 0.299 ± 0.127 | 0.226 ± 0.117 | 0.163 ± 0.049 |
|  | **DTO** | 0.253 ± 0.116 | 0.237 ± 0.068 | 0.283 ± 0.132 | 0.137 ± 0.089 | 0.184 ± 0.086 |
|  | **NDTO** | 0.241 ± 0.122 | 0.240 ± 0.086 | 0.234 ± 0.110 | 0.159 ± 0.099 | 0.227 ± 0.114 |
| **Non-Dominant  Knee Sagittal** | **DHS** | 0.352 ± 0.149 | 0.457 ± 0.231 | 0.200 ± 0.100 | 0.172 ± 0.071 | 0.246 ± 0.135 |
|  | **NDHS** | 0.230 ± 0.095 | 0.237 ± 0.099 | 0.320 ± 0.157 | 0.628 ± 0.185 | 0.425 ± 0.128 |
|  | **DTO** | 0.201 ± 0.083 | 0.249 ± 0.117 | 0.271 ± 0.171 | 0.523 ± 0.160 | 0.356 ± 0.146 |
|  | **NDTO** | 0.262 ± 0.120 | 0.322 ± 0.193 | 0.172 ± 0.109 | 0.137 ± 0.088 | 0.166 ± 0.080 |
| **Dominant  Ankle Sagittal** | **DHS** | 0.334 ± 0.149 | 0.302 ± 0.210 | 0.220 ± 0.113 | 0.147 ± 0.086 | 0.223 ± 0.110 |
|  | **NDHS** | 0.201 ± 0.083 | 0.195 ± 0.082 | 0.141 ± 0.067 | 0.134 ± 0.088 | 0.211 ± 0.149 |
|  | **DTO** | 0.256 ± 0.073 | 0.285 ± 0.109 | 0.309 ± 0.113 | 0.271 ± 0.150 | 0.350 ± 0.131 |
|  | **NDTO** | 0.341 ± 0.125 | 0.278 ± 0.144 | 0.198 ± 0.089 | 0.300 ± 0.070 | 0.230 ± 0.085 |
| **Dominant  Ankle Frontal** | **DHS** | 0.193 ± 0.105 | 0.167 ± 0.080 | 0.208 ± 0.073 | 0.086 ± 0.086 | 0.115 ± 0.061 |
|  | **NDHS** | 0.156 ± 0.062 | 0.132 ± 0.055 | 0.159 ± 0.093 | 0.093 ± 0.050 | 0.124 ± 0.050 |
|  | **DTO** | 0.144 ± 0.073 | 0.136 ± 0.081 | 0.222 ± 0.090 | 0.149 ± 0.079 | 0.164 ± 0.058 |
|  | **NDTO** | 0.197 ± 0.096 | 0.271 ± 0.144 | 0.280 ± 0.128 | 0.117 ± 0.054 | 0.088 ± 0.062 |
| **Dominant  Ankle Transverse** | **DHS** | 0.104 ± 0.060 | 0.154 ± 0.093 | 0.123 ± 0.098 | 0.045 ± 0.026 | 0.081 ± 0.041 |
|  | **NDHS** | 0.087 ± 0.038 | 0.078 ± 0.041 | 0.107 ± 0.041 | 0.063 ± 0.035 | 0.091 ± 0.042 |
|  | **DTO** | 0.098 ± 0.046 | 0.085 ± 0.043 | 0.167 ± 0.058 | 0.107 ± 0.034 | 0.123 ± 0.053 |
|  | **NDTO** | 0.131 ± 0.061 | 0.119 ± 0.032 | 0.148 ± 0.093 | 0.074 ± 0.025 | 0.078 ± 0.033 |
| **Dominant  Hip Sagittal** | **DHS** | 0.221 ± 0.081 | 0.163 ± 0.094 | 0.223 ± 0.099 | 0.251 ± 0.079 | 0.253 ± 0.064 |
|  | **NDHS** | 0.278 ± 0.107 | 0.231 ± 0.118 | 0.197 ± 0.082 | 0.229 ± 0.146 | 0.320 ± 0.125 |
|  | **DTO** | 0.256 ± 0.134 | 0.270 ± 0.132 | 0.178 ± 0.095 | 0.207 ± 0.128 | 0.268 ± 0.133 |
|  | **NDTO** | 0.205 ± 0.098 | 0.193 ± 0.079 | 0.266 ± 0.090 | 0.309 ± 0.105 | 0.295 ± 0.155 |
| **Dominant  Hip Frontal** | **DHS** | 0.117 ± 0.058 | 0.134 ± 0.038 | 0.172 ± 0.074 | 0.108 ± 0.044 | 0.146 ± 0.062 |
|  | **NDHS** | 0.150 ± 0.069 | 0.172 ± 0.093 | 0.201 ± 0.106 | 0.103 ± 0.064 | 0.165 ± 0.081 |
|  | **DTO** | 0.161 ± 0.049 | 0.149 ± 0.046 | 0.153 ± 0.081 | 0.094 ± 0.040 | 0.146 ± 0.063 |
|  | **NDTO** | 0.112 ± 0.055 | 0.141 ± 0.066 | 0.128 ± 0.077 | 0.126 ± 0.057 | 0.139 ± 0.080 |
| **Dominant  Hip Transverse** | **DHS** | 0.287 ± 0.116 | 0.184 ± 0.071 | 0.350 ± 0.154 | 0.178 ± 0.092 | 0.194 ± 0.083 |
|  | **NDHS** | 0.215 ± 0.133 | 0.257 ± 0.139 | 0.310 ± 0.171 | 0.224 ± 0.115 | 0.281 ± 0.099 |
|  | **DTO** | 0.260 ± 0.129 | 0.218 ± 0.093 | 0.252 ± 0.150 | 0.202 ± 0.110 | 0.225 ± 0.081 |
|  | **NDTO** | 0.283 ± 0.134 | 0.232 ± 0.079 | 0.264 ± 0.119 | 0.179 ± 0.085 | 0.217 ± 0.078 |
| **Dominant  Knee Sagittal** | **DHS** | 0.241 ± 0.121 | 0.174 ± 0.123 | 0.310 ± 0.189 | 0.703 ± 0.190 | 0.445 ± 0.183 |
|  | **NDHS** | 0.365 ± 0.127 | 0.413 ± 0.146 | 0.235 ± 0.119 | 0.179 ± 0.092 | 0.287 ± 0.107 |
|  | **NDTO** | 0.293 ± 0.099 | 0.332 ± 0.123 | 0.190 ± 0.082 | 0.127 ± 0.079 | 0.192 ± 0.083 |
|  | **DTO** | 0.210 ± 0.084 | 0.201 ± 0.094 | 0.303 ± 0.182 | 0.539 ± 0.175 | 0.349 ± 0.063 |

**Figure S3. Mean and standard error of the dominant knee and non-dominant knee’s sagittal joint angle variability, respectively.** Each bar represents the mean value of the joint angle variability at Poincaré sections across slope conditions, and the error bars represent the standard error.

**Table S4. Post hoc pairwise comparison results regarding the effect of Poincaré sections on the maximum Floquet multipliers.**

| **Comparison** | |  | | | | |
| --- | --- | --- | --- | --- | --- | --- |
| **Poincaré Section** | **Poincaré Section** | **Mean Difference** | **SE** | **df** | **t** | $\boldsymbol{p}_{\boldsymbol{bonferroni}}$ |
| **DHS** | **NDHS** | 0.01337 | 0.00672 | 12.0 | 1.989 | 0.420 |
|  | **DTO** | 0.02425 | 0.00529 | 12.0 | 4.585 | 0.004 |
|  | **NDTO** | 0.02209 | 0.00729 | 12.0 | 3.031 | 0.063 |
| **NDHS** | **DTO** | 0.01087 | 0.00812 | 12.0 | 1.340 | 1.000 |
|  | **NDTO** | 0.00872 | 0.00808 | 12.0 | 1.079 | 1.000 |
| **DTO** | **NDTO** | -0.00215 | 0.00736 | 12.0 | -0.293 | 1.000 |
