# Supplementary material for: The adaptive role of the knee joint in maintaining the orbital stability during slope walking
Source: R Soc Open Sci. 2026 Sep 9;13(9):rsos252138. doi: 10.1098/rsos.252138 (PMC13561039; doi:10.1098/rsos.252138)
Supplement: Supplementary file 1 [file rsos.252138.zip › rsos.252138/rsos252138_si_002.pdf]

Supplementary

Table S1. Mean and standard deviation of range of motion (ROM) and minimum to maximum angles in lower limb joints aggregated from strides defined by subsequent dominant leg heel strikes across slope conditions.

***Mean±SD (Min – Max)**

	Slope				
**Joint ROMs**	**-12°**	**-6°**	**0°**	**6°**	**12°**
**Non-Dominant Ankle Sagittal**	33.08 ± 2.52 (-13.81 – 19.26)	31.36 ± 4.89 (-12.61 – 18.75)	31.58 ± 3.71 (-14.49 – 17.09)	36.04 ± 4.41 (-15.45 – 20.58)	39.32 ± 5.00 (-13.69 – 25.63)
**Non-Dominant Ankle Frontal**	14.18 ± 2.70 (-12.51 – 1.66)	13.94 ± 3.98 (-12.33 – 1.61)	11.71 ± 3.24 (-11.25 – 0.47)	9.19 ± 1.61 (-10.30 – -1.10)	8.73 ± 2.17 (-10.99 – -2.26)
**Non-Dominant Ankle Transverse**	6.95 ± 1.91 (-10.30 – -3.35)	7.71 ± 2.36 (-10.34 – -2.62)	6.71 ± 1.47 (-9.93 – -3.21)	7.19 ± 1.82 (-8.89 – -1.70)	7.68 ± 2.32 (-8.17 – -0.49)
**Non-Dominant Hip Sagittal**	19.20 ± 2.75 (11.17 – 30.36)	28.07 ± 4.02 (3.71 – 31.79)	37.90 ± 3.13 (-1.88 – 36.02)	48.22 ± 3.44 (-1.15 – 47.07)	59.38 ± 5.41 (-0.02 – 59.36)
**Non-Dominant Hip Frontal**	11.43 ± 2.74 (-4.37 – 7.07)	12.48 ± 2.59 (-5.25 – 7.24)	12.14 ± 2.42 (-4.70 – 7.44)	11.58 ± 2.53 (-4.95 – 6.63)	12.04 ± 3.21 (-5.25 – 6.79)
**Non-Dominant Hip Transverse**	12.63 ± 2.76 (-2.65 – 9.99)	12.50 ± 2.93 (-2.76 – 9.74)	11.53 ± 2.06 (-1.55 – 9.98)	10.64 ± 1.89 (-2.25 – 8.39)	11.02 ± 1.98 (-3.21 – 7.82)
**Non-Dominant Knee Sagittal**	73.35 ± 4.50 (-78.79 – -5.44)	68.23 ± 5.42 (-71.85 – -3.63)	65.31 ± 4.24 (-69.11 – -3.80)	55.46 ± 4.28 (-66.29 – -10.83)	56.59 ± 5.26 (-67.27 – -10.68)
**Dominant Ankle Sagittal**	32.43 ± 3.24 (-13.08 – 19.35)	29.90 ± 3.80 (-11.53 – 18.37)	31.71 ± 3.37 (-15.38 – 16.32)	35.45 ± 3.56 (-15.48 – 19.97)	39.16 ± 4.40 (-13.55 – 25.61)

Joint ROMs	Slope				
	**-12°**	**-6°**	**0°**	**6°**	**12°**
**Dominant Ankle Frontal**	16.57 ± 3.53 (-1.29 – 15.28)	15.94 ± 3.84 (-0.94 – 14.99)	12.93 ± 3.56 (0.44 – 13.37)	9.54 ± 1.39 (2.29 – 11.83)	8.80 ± 1.26 (3.67 – 12.47)
**Dominant Ankle Transverse**	7.04 ± 1.83 (0.36 – 7.40)	7.42 ± 1.53 (-0.03 – 7.39)	6.77 ± 1.71 (-0.12 – 6.66)	7.21 ± 1.92 (-1.68 – 5.53)	8.09 ± 2.09 (-3.30 – 4.79)
**Dominant Hip Sagittal**	19.08 ± 2.51 (10.34 – 29.42)	28.28 ± 3.36 (2.55 – 30.83)	37.38 ± 2.69 (-2.33 – 35.05)	47.97 ± 2.96 (-1.57 – 46.40)	59.10 ± 4.92 (0.04 – 59.14)
**Dominant Hip Frontal**	11.52 ± 2.23 (-3.68 – 7.84)	12.53 ± 2.67 (-4.54 – 7.99)	12.77 ± 3.12 (-4.99 – 7.78)	12.75 ± 3.01 (-4.55 – 8.21)	13.48 ± 3.50 (-4.99 – 8.50)
**Dominant Hip Transverse**	12.74 ± 3.10 (-8.76 – 3.98)	12.74 ± 2.67 (-9.04 – 3.70)	11.58 ± 2.30 (-8.88 – 2.70)	10.90 ± 1.47 (-7.47 – 3.42)	11.18 ± 1.55 (-7.01 – 4.17)
**Dominant Knee Sagittal**	72.57 ± 4.60 (-77.13 – -4.56)	68.02 ± 5.05 (-70.95 – -2.93)	65.56 ± 4.64 (-68.55 – -2.99)	55.27 ± 5.03 (-65.56 – -10.29)	55.09 ± 4.97 (-66.46 – -11.38)

Table S2. Mean and standard deviation of 14-dimensional eigenvector component magnitudes corresponding to the maximum Floquet multiplier at each Poincaré section across slope conditions.

Eigenvector Component	Poincaré Section	Slope				
		**-12°**	**-6°**	**0°**	**6°**	**12°**
**Non-Dominant Ankle Sagittal**	**DHS**	0.156 ± 0.061	0.235 ± 0.091	0.117 ± 0.054	0.130 ± 0.061	0.165 ± 0.125
	**NDHS**	0.276 ± 0.106	0.258 ± 0.194	0.211 ± 0.112	0.169 ± 0.081	0.203 ± 0.092
	**DTO**	0.370 ± 0.153	0.300 ± 0.152	0.194 ± 0.076	0.297 ± 0.104	0.260 ± 0.112
	**NDTO**	0.272 ± 0.095	0.276 ± 0.122	0.250 ± 0.106	0.294 ± 0.117	0.351 ± 0.157

Eigenvector Component	Poincaré Section	Slope				
		**-12°**	**-6°**	**0°**	**6°**	**12°**
**Non-Dominant Ankle Frontal**	**DHS**	0.109 ± 0.039	0.122 ± 0.045	0.163 ± 0.090	0.075 ± 0.031	0.128 ± 0.059
	**NDHS**	0.174 ± 0.060	0.187 ± 0.077	0.178 ± 0.117	0.098 ± 0.052	0.102 ± 0.045
	**DTO**	0.189 ± 0.103	0.212 ± 0.110	0.193 ± 0.076	0.091 ± 0.053	0.084 ± 0.043
	**NDTO**	0.147 ± 0.083	0.145 ± 0.054	0.178 ± 0.112	0.103 ± 0.053	0.131 ± 0.076
**Non-Dominant Ankle Transverse**	**DHS**	0.061 ± 0.032	0.062 ± 0.030	0.118 ± 0.052	0.048 ± 0.028	0.085 ± 0.043
	**NDHS**	0.107 ± 0.049	0.144 ± 0.075	0.121 ± 0.072	0.057 ± 0.036	0.081 ± 0.046
	**DTO**	0.104 ± 0.050	0.147 ± 0.087	0.140 ± 0.053	0.074 ± 0.042	0.083 ± 0.047
	**NDTO**	0.083 ± 0.054	0.066 ± 0.034	0.104 ± 0.061	0.085 ± 0.041	0.118 ± 0.059
**Non-Dominant Hip Sagittal**	**DHS**	0.291 ± 0.110	0.245 ± 0.121	0.240 ± 0.080	0.229 ± 0.166	0.337 ± 0.160
	**NDHS**	0.259 ± 0.097	0.190 ± 0.095	0.267 ± 0.078	0.250 ± 0.112	0.242 ± 0.122
	**DTO**	0.229 ± 0.097	0.224 ± 0.135	0.251 ± 0.108	0.287 ± 0.075	0.304 ± 0.123
	**NDTO**	0.250 ± 0.125	0.252 ± 0.128	0.224 ± 0.070	0.235 ± 0.125	0.300 ± 0.164
**Non-Dominant Hip Frontal**	**DHS**	0.154 ± 0.079	0.151 ± 0.075	0.192 ± 0.131	0.098 ± 0.059	0.142 ± 0.089
	**NDHS**	0.154 ± 0.070	0.151 ± 0.088	0.125 ± 0.048	0.115 ± 0.071	0.166 ± 0.050
	**DTO**	0.134 ± 0.076	0.113 ± 0.048	0.163 ± 0.079	0.126 ± 0.071	0.183 ± 0.058
	**NDTO**	0.156 ± 0.080	0.170 ± 0.059	0.193 ± 0.095	0.120 ± 0.051	0.130 ± 0.067
**Non-Dominant Hip Transverse**	**DHS**	0.241 ± 0.138	0.249 ± 0.110	0.273 ± 0.113	0.175 ± 0.070	0.224 ± 0.060
	**NDHS**	0.290 ± 0.125	0.213 ± 0.106	0.299 ± 0.127	0.226 ± 0.117	0.163 ± 0.049
	**DTO**	0.253 ± 0.116	0.237 ± 0.068	0.283 ± 0.132	0.137 ± 0.089	0.184 ± 0.086
	**NDTO**	0.241 ± 0.122	0.240 ± 0.086	0.234 ± 0.110	0.159 ± 0.099	0.227 ± 0.114
**Non-Dominant Knee Sagittal**	**DHS**	0.352 ± 0.149	0.457 ± 0.231	0.200 ± 0.100	0.172 ± 0.071	0.246 ± 0.135
	**NDHS**	0.230 ± 0.095	0.237 ± 0.099	0.320 ± 0.157	0.628 ± 0.185	0.425 ± 0.128
	**DTO**	0.201 ± 0.083	0.249 ± 0.117	0.271 ± 0.171	0.523 ± 0.160	0.356 ± 0.146
	**NDTO**	0.262 ± 0.120	0.322 ± 0.193	0.172 ± 0.109	0.137 ± 0.088	0.166 ± 0.080
**Dominant Ankle Sagittal**	**DHS**	0.334 ± 0.149	0.302 ± 0.210	0.220 ± 0.113	0.147 ± 0.086	0.223 ± 0.110
	**NDHS**	0.201 ± 0.083	0.195 ± 0.082	0.141 ± 0.067	0.134 ± 0.088	0.211 ± 0.149
	**DTO**	0.256 ± 0.073	0.285 ± 0.109	0.309 ± 0.113	0.271 ± 0.150	0.350 ± 0.131
	**NDTO**	0.341 ± 0.125	0.278 ± 0.144	0.198 ± 0.089	0.300 ± 0.070	0.230 ± 0.085

Eigenvector Component	Poincaré Section	Slope				
		-12°	-6°	0°	6°	12°
Dominant Ankle Frontal	DHS	0.193 ± 0.105	0.167 ± 0.080	0.208 ± 0.073	0.086 ± 0.086	0.115 ± 0.061
	NDHS	0.156 ± 0.062	0.132 ± 0.055	0.159 ± 0.093	0.093 ± 0.050	0.124 ± 0.050
	DTO	0.144 ± 0.073	0.136 ± 0.081	0.222 ± 0.090	0.149 ± 0.079	0.164 ± 0.058
	NDTO	0.197 ± 0.096	0.271 ± 0.144	0.280 ± 0.128	0.117 ± 0.054	0.088 ± 0.062
Dominant Ankle Transverse	DHS	0.104 ± 0.060	0.154 ± 0.093	0.123 ± 0.098	0.045 ± 0.026	0.081 ± 0.041
	NDHS	0.087 ± 0.038	0.078 ± 0.041	0.107 ± 0.041	0.063 ± 0.035	0.091 ± 0.042
	DTO	0.098 ± 0.046	0.085 ± 0.043	0.167 ± 0.058	0.107 ± 0.034	0.123 ± 0.053
	NDTO	0.131 ± 0.061	0.119 ± 0.032	0.148 ± 0.093	0.074 ± 0.025	0.078 ± 0.033
Dominant Hip Sagittal	DHS	0.221 ± 0.081	0.163 ± 0.094	0.223 ± 0.099	0.251 ± 0.079	0.253 ± 0.064
	NDHS	0.278 ± 0.107	0.231 ± 0.118	0.197 ± 0.082	0.229 ± 0.146	0.320 ± 0.125
	DTO	0.256 ± 0.134	0.270 ± 0.132	0.178 ± 0.095	0.207 ± 0.128	0.268 ± 0.133
	NDTO	0.205 ± 0.098	0.193 ± 0.079	0.266 ± 0.090	0.309 ± 0.105	0.295 ± 0.155
Dominant Hip Frontal	DHS	0.117 ± 0.058	0.134 ± 0.038	0.172 ± 0.074	0.108 ± 0.044	0.146 ± 0.062
	NDHS	0.150 ± 0.069	0.172 ± 0.093	0.201 ± 0.106	0.103 ± 0.064	0.165 ± 0.081
	DTO	0.161 ± 0.049	0.149 ± 0.046	0.153 ± 0.081	0.094 ± 0.040	0.146 ± 0.063
	NDTO	0.112 ± 0.055	0.141 ± 0.066	0.128 ± 0.077	0.126 ± 0.057	0.139 ± 0.080
Dominant Hip Transverse	DHS	0.287 ± 0.116	0.184 ± 0.071	0.350 ± 0.154	0.178 ± 0.092	0.194 ± 0.083
	NDHS	0.215 ± 0.133	0.257 ± 0.139	0.310 ± 0.171	0.224 ± 0.115	0.281 ± 0.099
	DTO	0.260 ± 0.129	0.218 ± 0.093	0.252 ± 0.150	0.202 ± 0.110	0.225 ± 0.081
	NDTO	0.283 ± 0.134	0.232 ± 0.079	0.264 ± 0.119	0.179 ± 0.085	0.217 ± 0.078
Dominant Knee Sagittal	DHS	0.241 ± 0.121	0.174 ± 0.123	0.310 ± 0.189	0.703 ± 0.190	0.445 ± 0.183
	NDHS	0.365 ± 0.127	0.413 ± 0.146	0.235 ± 0.119	0.179 ± 0.092	0.287 ± 0.107
	NDTO	0.293 ± 0.099	0.332 ± 0.123	0.190 ± 0.082	0.127 ± 0.079	0.192 ± 0.083
	DTO	0.210 ± 0.084	0.201 ± 0.094	0.303 ± 0.182	0.539 ± 0.175	0.349 ± 0.063

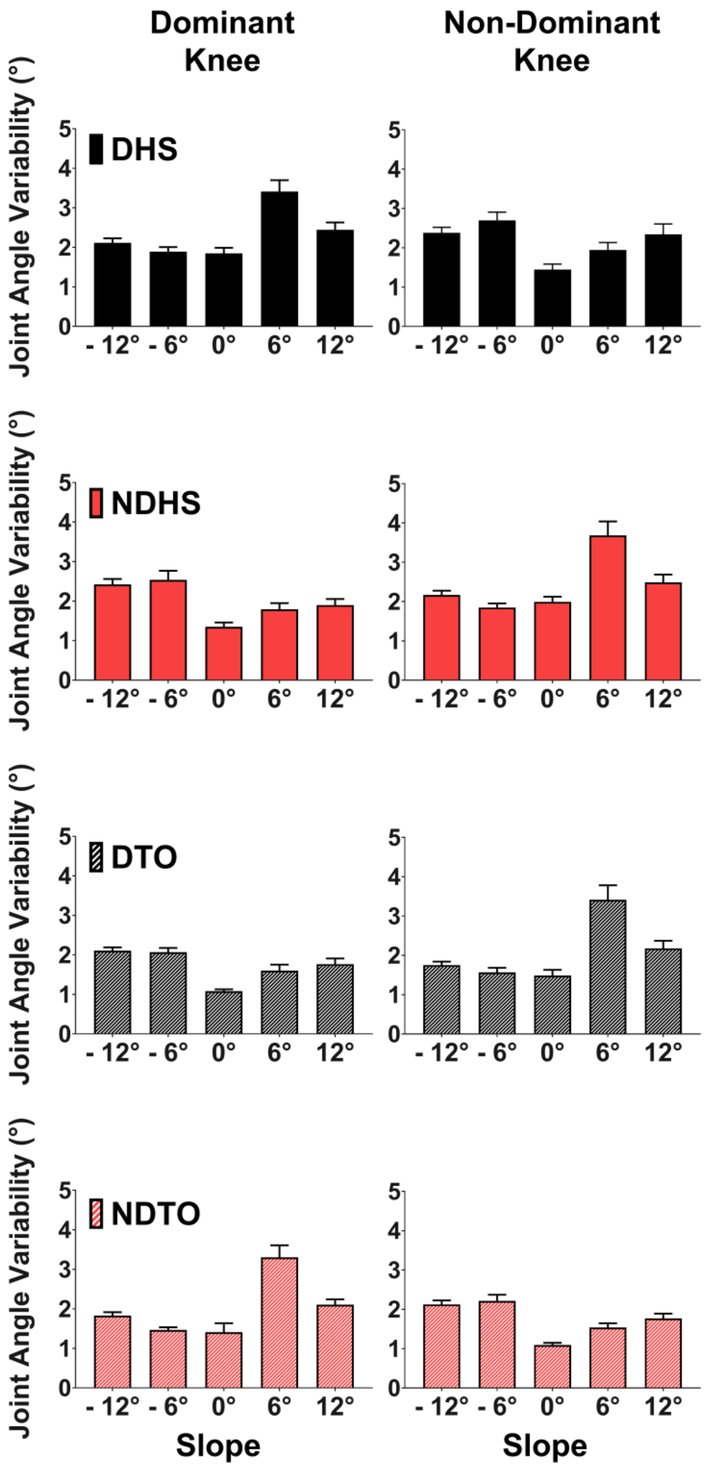

Figure S3. Mean and standard error of the dominant knee and non-dominant knee's sagittal joint angle variability, respectively. Each bar represents the mean value of the joint angle variability at Poincaré sections across slope conditions, and the error bars represent the standard error.

Table S4. Post hoc pairwise comparison results regarding the effect of Poincaré sections on the maximum Floquet multipliers.

Comparison						
**Poincaré Section**	**Poincaré Section**	**Mean Difference**	**SE**	**df**	**t**	$p_{bonferroni}$
**DHS**	**NDHS**	0.01337	0.00672	12.0	1.989	0.420
	**DTO**	0.02425	0.00529	12.0	4.585	0.004
	**NDTO**	0.02209	0.00729	12.0	3.031	0.063
**NDHS**	**DTO**	0.01087	0.00812	12.0	1.340	1.000
	**NDTO**	0.00872	0.00808	12.0	1.079	1.000
**DTO**	**NDTO**	-0.00215	0.00736	12.0	-0.293	1.000
